# Association between mukbang and cookbang viewing and body image perception and BMI in adolescents

**DOI:** 10.1186/s41043-024-00552-0

**Published:** 2024-05-09

**Authors:** Hyesun Jeong, Eunyoung Lee, Gyumin Han

**Affiliations:** 1 Department of Nursing, Daedong College, Busan, Republic of Korea; 2 College of Nursing, Catholic University of Pusan, Busan, Republic of Korea; 3 Research Institute of Nursing Science, Pusan National University, 49, Busandaehak-ro, Mulgeum-eup, Yangsan-si, Gyeongsangnam-do 50612 Republic of Korea

**Keywords:** Adolescent, Body image, Body Mass Index, Cookbang, Media exposure, Mukbang

## Abstract

**Background:**

Adolescence is a critical period for establishing healthy eating habits and weight management, essential for preventing obesity and promoting overall health. This study investigates the impact of mukbang and cookbang—popular online broadcasts in Korea that feature excessive consumption of food—on the dietary habits and body image perception of Korean adolescents. With digital media, especially platforms like YouTube, becoming an integral part of daily life, these broadcasts have the potential to significantly influence adolescent health behaviors.

**Methods:**

Employing data from the 18th Korea Youth Risk Behavior Web-based Survey (2022), this descriptive survey research explores the relationship between watching mukbang and cookbang and various health-related factors among adolescents. The survey’s comprehensive dataset provided a unique opportunity to examine this association in a population that is increasingly exposed to digital media content. The analysis focused on the frequency of watching mukbang and cookbang, their impact on eating habits, body mass index (BMI), body shape perception, and body image distortion among adolescents.

**Results:**

The results revealed a significant engagement with mukbang and cookbang among adolescents, with notable gender differences in viewing habits and effects. Increased frequency of viewing was associated with negative impacts on eating habits and body image perception. Furthermore, psychological factors such as stress levels and sleep quality emerged as significant predictors of the frequency of watching these broadcasts.

**Conclusions:**

This study highlights the need for further investigation into the causal relationships between mukbang and cookbang viewership and adolescent health outcomes. The findings suggest the importance of developing targeted interventions to mitigate the negative influences of such content on adolescents’ eating habits and body perceptions. Given the widespread popularity of these broadcasts, it is crucial to address their potential health implications through public health strategies, educational content, and policy development aimed at promoting healthier lifestyles among adolescents.

## Background

Adolescence is a critical period of rapid physical and psychological development, where nutrition management and weight control play significant roles. Adequate nutritional intake during adolescence supports healthy growth and development, and is known to reduce the risk of obesity and related diseases [1]. Furthermore, healthy eating habits in adolescence are evaluated as the foundation for a long-term healthy lifestyle [2]. Moreover, dietary habits during adolescence can have long-term effects on adult health [3], highlighting the importance of research on adolescents’ dietary habits.

The use of media is deeply embedded in the daily lives of adolescents in modern society, significantly impacting important health-related aspects such as eating habits, weight management, and body image perception [4]. Strict control and censorship of traditional media content limit exposure to sensational or inappropriate material, and related research has provided insights into how adolescents perceive and decide on food choices and eating behaviors, as well as how societal standards for weight and body image are formed. However, with the advent of platforms like YouTube, where individuals can freely produce and share content, adolescents are increasingly exposed to various forms of media. This shift has expanded the range of information accessible to adolescents but has also increased the risk of exposure to inappropriate content. Despite these changes, there is a relative lack of research specifically addressing the impact of these media forms on adolescent behavior.

One of the most popular content trends on online platforms in recent years is “mukbang” and “cookbang,” broadcasts that focus on eating or cooking [5]. “Mukbang” is a portmanteau of the Korean words for “eating” and “broadcast,” while “cookbang” refers to online cooking shows, both of which originated in Korea and have spread widely across various international online platforms like YouTube [6]. While mukbang and cookbang content can help alleviate the loneliness of those eating alone [7], the majority of these broadcasts involve the consumption of excessive amounts of high-calorie meals, significantly impacting adolescents’ eating habits [8]. According to a study on the patterns of mukbang and cookbang consumption among Korean adolescents, 39.6% reported watching mukbang or cookbang at least once a week, with a higher frequency of viewing associated with poorer eating habits (9). However, there is a lack of preliminary research exploring the association between watching mukbang and cookbang and various risk factors among adolescents, including not only eating habits but also body image perception and inappropriate weight control measures.

Adolescents are particularly vulnerable to the influences of media, which can significantly shape their dietary behaviors and health perceptions. Accordingly, this study is dedicated to systematically examining the prevalence of mukbang (eating broadcasts) and cookbang (cooking broadcasts) viewing among Korean adolescents, and identifying the factors influencing this phenomenon. The research specifically investigates a comprehensive set of variables, including socio-demographic (such as age, gender, economic status), psychological (such as stress levels and sleep quality), and body image-related factors (such as self-perceived body image and actual body mass index). Importantly, the study acknowledges the potential variability in dietary habits and body image concerns across genders. Therefore, it conducts separate analyses for males and females to uncover gender-specific dynamics. This gender-focused analysis is pivotal as it provides critical insights that could guide the development of gender-specific health promotion programs aimed at more effectively addressing the unique needs of each group.

## Methods

### Aim and study design

This study is a descriptive survey research that attempts to clarify the relationship between the consumption of mukbang and cookbang and factors related to body image perception among Korean adolescents. It is a secondary analysis study utilizing the raw data from the 18th Korea Youth Risk Behavior Web-based Survey (2022) conducted by the Korea Disease Control and Prevention Agency. This dataset was selected due to its comprehensive and relevant data on adolescent health behaviors, providing a robust foundation for analyzing the specific media influences on body image.

### Setting and sample

The Korea Youth Risk Behavior Web-based Survey is an online self-administered survey conducted annually since 2005 by the Ministry of Education, Ministry of Health and Welfare, and Korea Disease Control and Prevention Agency. It aims to understand the health behaviors of Korean adolescents from the first year of middle school to the third year of high school nationwide. For this study, raw data was requested and obtained through the youth health behavior online survey website (http://yhs.cdc.go.kr) in accordance with the Korea Disease Control and Prevention Agency’s regulations for raw data release and management. The 18th survey (2022) statistical data was based on a stratified cluster sample extracted in August 2022, using the national population of middle and high school students as the sampling population. The primary sampling units were selected through systematic sampling from a list of schools categorized by region, school type (middle schools, general high schools, and specialized high schools), considering geographical accessibility, number of schools, population size, and living environment as stratification variables. The secondary sampling units were classes, with one class randomly selected from each grade within the selected schools. The survey was conducted anonymously online among students from 800 sampled schools, with 51,850 participants out of 56,213, showing a participation rate of 92.4%. For the analysis concerning body image distortion, data from 50,455 respondents were used after excluding those with missing values for height and weight.

### Measurements

#### Demographic and sociological characteristics

Gender was categorized into ‘male’ and ‘female’ as per the original response scale. School type was originally categorized into six grades across middle and high schools (1st to 3rd year for both), which was then reclassified into two categories, middle school and high school, to observe differences based on school level. Subjective academic performance and economic status were reclassified from the original response scale for comparison with prior studies: ‘high’ remained ‘high’, both ‘above average’ and ‘average’ were grouped into ‘medium’, and ‘below average’ and ‘low’ were grouped into ‘low’, resulting in three categories. Current living situation was reclassified into three categories: living with family, not living with family (including living in a relative’s house, boarding, or independently), and living in institutional settings such as childcare facilities or orphanages. The degree of fatigue recovery from sleep was categorized based on the question “In the past 7 days, do you think the amount of sleep you got was sufficient for fatigue recovery?” into ‘sufficient’ (very sufficient, sufficient), ‘average’ (so-so), and ‘insufficient’ (not sufficient, not at all sufficient). Perception of usual stress was reclassified from the original response scale ‘feel very stressed’ and ‘feel stressed’ to ‘high’, ‘feel a little stressed’ to ‘medium’, and ‘do not feel much stressed’ and ‘do not feel stressed at all’ to ‘low’.

### Watching mukbang and cookbang

The frequency of watching mukbang (eating broadcasts) and cookbang (cooking broadcasts) over the past 12 months was assessed with the question, “How often have you watched mukbang and cookbang in the last 12 months?” The responses were reclassified into four categories: ‘Never watched’, ‘Occasionally watched (less than once a month, 1 to 3 times a month)’, ‘Frequently watched (1–2 times a week, 3–4 times a week)’, and ‘Almost daily watched (5–6 times a week, every day)’.

### The impact of watching mukbang and cookbang on eating habits

The impact of watching mukbang and cookbang on eating habits was assessed with the question, “What is the biggest impact that watching mukbang (eating broadcasts) and cookbang (cooking broadcasts) has on your eating habits?” The original response scale was used as is, with options ‘No impact’, ‘Eating faster’, ‘Eating more’, ‘Following the food eaten or cooked in mukbang or cookbang’, ‘Eating stimulating foods such as spicy, salty, sweet, or rich foods’. Respondents who do not watch mukbang and cookbang were categorized as ‘Did not respond’.

#### Variables related to body image distortion

##### Body mass index (BMI)

BMI (Body Mass Index, weight in kg/[height in m]^2^) was calculated using self-reported height and weight. Using the 2017 Korean growth charts for children and adolescents, BMI was categorized into underweight (less than the 5th percentile), normal weight (5th-84th percentile), overweight (85th-94th percentile), and obese (95th percentile or above) groups [9].

### Body perception and body image distortion

Body shape perception was assessed with a 5-point Likert scale question and re-categorized into ‘Thin’ (‘Very thin’, ‘Slightly thin’), ‘Normal’ (‘Normal’), ‘Obese’ (‘Slightly overweight’, ‘Very overweight’). Body perception analysis compared the weight status categorized by BMI with the participant’s perceived weight status: an equal perception when the participant’s perception matched their BMI category, over-perception when individuals of normal weight perceived themselves as ‘Obese’ or individuals of underweight perceived themselves as ‘Normal’ or ‘Obese’, and under-perception when individuals of normal weight perceived themselves as ‘Thin’, or individuals of obese category perceived themselves as ‘Normal’ or ‘Thin’. Body image distortion was identified by distinguishing between over-perception or under-perception (indicating presence of body image distortion) and equal perception (indicating no body image distortion) [10].

### Ethical considerations

This study was conducted after receiving an exemption from review by the Public Institutional Review Board designated by Ministry of Health and Welfare (P01-202401-01-040).

### Data analysis

Since the sample for the Youth Health Behavior Online Survey was drawn using a complex sample design method, the analysis was conducted in accordance with the Korea Disease Control and Prevention Agency’s guidelines for analyzing complex sample design data. This involved using stratification variables (strata), cluster variables (cluster), weights (w), and the finite population correction factor in the analysis.

Descriptive statistics for the frequency of watching mukbang and cookbang, the impact of cookbang watching on eating habits, and general characteristics were analyzed by gender using unweighted frequencies and weighted percentages. To compare characteristics according to the frequency of watching mukbang, the Rao-Scott χ2 test was conducted. Furthermore, we utilized hierarchical logistic regression to analyze the influences on adolescents’ consumption of mukbang and cookbang. The model sequentially adjusted for different categories of variables to explore their independent and cumulative impacts. Initially, sociodemographic characteristics such as grade of school, academic performance, socioeconomic status, and living type were incorporated to establish a baseline. Subsequent layers introduced psychological variables including perceived fatigue recovery by sleep and perceived stress. The final layer added body image-related variables, specifically BMI and body image perception. This structured approach allowed us to delineate the direct and interactive effects of these variables on the viewing habits of adolescents, providing a comprehensive understanding of the influences shaping media consumption.

## Results

### The frequency of watching mukbang and cookbang and their impact on eating habits among male and female adolescents

As shown in Table 1, the frequency of watching mukbang and cookbang among adolescents showed that 9,289 male students (37%) reported never watching, compared to 5,140 female students (21.5%), indicating a higher percentage among male students. Conversely, daily viewers were more common among female students, with 2,393 (9.5%) compared to 1,854 male students (7%) (χ^2^ = 235.21, *p* < .001).

**Table 1:** Characteristics of the mukbang and cukbang watching (*n* = 50,455)

	Total (*n* = 50,455)	Male (*n* = 25,751)	Female (*n* = 24,704)	χ^2^ (*p*)
Frequency of watching				
Not watching	14,429 (29.5)	9,289 (37.0)	5,140 (21.5)	235.21 (< 0.001)
Less than once a month	5,170 (10.3)	2,682 (10.6)	2,488 (10.0)	
More than 1 to 3 times a month	9,343 (18.4)	4,164 (16.0)	5,179 (20.9)	
1–2 times a week	8,953 (17.5)	4,208 (16.0)	4,745 (19.2)	
3–4 times a week	5,818 (11.3)	2,538 (9.6)	3,280 (13.1)	
5–6 times a week	2,495 (4.8)	1,016 (3.9)	1,479 (5.8)	
Everyday	4,247 (8.2)	1,854 (7.0)	2,393 (9.5)	

### General characteristics of adolescents by frequency of watching mukbang and cookbang

Analysis of general characteristics related to the frequency of watching mukbang and cookbang among male students revealed statistically significant differences in all areas except living situation, with notable differences in academic performance (χ^2^ = 51.81, *p* < .001) and perceived stress (χ^2^ = 19.05, *p* < .001) (Table 2). Similarly, among female students, significant differences were observed in all areas except living situation, especially in academic performance (χ^2^ = 47.43, *p* < .001) and perceived stress (χ^2^ = 22.39, *p* < .001) (Table 3).

**Table 2:** Characteristics of the male adolescents by number of mukbang and cookbang watching (*n* = 25,751)

Variables	Categories	Male				
		None	Rare	Often	Almost everyday	χ2 (*p*)
Grade of school	Middle school	4,914(50.8)	3,827(53.8)	3,575(50.4)	1,538(51.6)	5.10 (0.002)
	High school-	4,375 (49.2)	3,019 (46.2)	3,171 (49.6)	1,332 (48.4)	
Academic performance	High	1,662 (17.9)	1,011 (15.1)	814 (11.8)	320 (11.2)	51.81 (< 0.001)
	Middle	5,089 (55.2)	3,787 (55.7)	3,585 (52.9)	1,418 (49.3)	
	Low	2,537 (26.9)	2,048 (29.2)	2,346 (35.2)	1,132 (39.5)	
Socioeconomic status	High	1,338 (14.8)	814 (12.2)	773 (11.5)	381 (13.9)	11.68 (< 0.001)
	Middle	7,029 (75.8)	5,321 (78.1)	5,230 (77.6)	2,124 (73.5)	
	Low	921 (9.4)	711 (9.7)	742 (10.9)	365 (12.7)	
Living type	With family	8,788 (95.3)	6,466 (95.1)	6,343 (95.0)	2,688 (94.3)	1.95 (0.099)
	Boarding house/relatives	88 (0.9)	92 (1.3)	92 (1.4)	40 (1.4)	
	Dorms	411 (3.8)	288 (3.6)	309 (3.6)	142 (4.3)	
Perceived fatigue recovery by sleep	Enough	2,765 (29.4)	1,801 (25.4)	1,688 (24.6)	698 (23.9)	16.23 (< 0.001)
	Moderate	3,001 (31.9)	2,430 (35.7)	2,427 (36.2)	912 (31.5)	
	Not enough	3,523 (38.7)	2,615 (38.9)	2,631 (39.2)	1,260 (44.6)	
Perceived Stress	High	3,153 (34.3)	2,343 (34.5)	2,425 (36.0)	1,207 (42.5)	19.05 (< 0.001)
	Moderate	3,935 (42.5)	3,122 (45.8)	3,053 (45.2)	1,162 (40.8)	
	Low	2,201 (23.2)	1,381 (19.6)	1,268 (18.7)	501 (16.7)	

**Table 3:** Characteristics of the female adolescents by number of mukbang and cookbang watching (*n* = 24,704)

Variables	Categories	Female				
		None	Rare	Often	Almost everyday	χ2 (*p*)
Grade of school	Middle school	2,769 (51.3)	4,369 (54.4)	4,294 (51.1)	2,024 (49.7)	7.32 (< 0.001)
	High school-	2,371(48.7)	3,298 (45.6)	3,731 (48.9)	1,848 (50.3)	
Academic performance	High	849 (16.5)	959 (12.5)	813 (10.1)	311 (8.1)	47.43 (< 0.001)
	Middle	2,978 (58.4)	4,471 (58.4)	4,647 (58.0)	2,072 (53.7)	
	Low	1,313 (25.1)	2,237 (29.2)	2,565 (31.9)	1,489 (38.2)	
Socioeconomic status	High	611 (12.0)	769 (10.5)	708 (9.0)	353 (9.4)	7.43 (< 0.001)
	Middle	3,954 (77.3)	6,080 (79.4)	6,428 (80.7)	3,007 (78.0)	
	Low	575 (10.7)	818 (10.1)	889 (10.3)	512 (12.6)	
Living type	With family	4,909 (96.2)	7,376 (96.8)	7,712 (96.8)	3,704 (96.3)	1.07 (0.377)
	Boarding house/relatives	43 (0.8)	70 (0.8)	57 (0.7)	39 (0.9)	
	Dorms	188 (3.0)	221 (2.4)	256 (2.5)	129 (2.8)	
Perceived fatigue recovery by sleep	Enough	1,057 (20.3)	1,391 (17.9)	1,278 (16.0)	627 (15.9)	14.21 (< 0.001)
	Moderate	1,485 (28.4)	2,448 (31.5)	2,514 (31.3)	1,049 (26.8)	
	Not enough	2,598 (51.3)	3,828 (50.6)	4,233 (52.7)	2,196 (57.3)	
Perceived Stress	High	2,269 (45.1)	3,417 (44.6)	3,771 (46.7)	2,111 (54.2)	22.39 (< 0.001)
	Moderate	2,060 (39.6)	3,238 (42.3)	3,306 (41.4)	1,317 (34.3)	
	Low	811 (15.3)	1,012 (13.1)	948 (11.9)	444 (11.5)	

### The impact of watching mukbang and cookbang on eating habits by frequency among male and female adolescents

The impacts of mukbang and cookbang on eating habits among adolescents are summarized in Table 4. Excluding those who reported never watching mukbang and cookbang (14,429), the analysis of 36,026 respondents showed that 4,933 male students (71.8%) who rarely watched reported no impact on their eating habits. In contrast, a significant impact was noted among those who frequently watched, with 1,182 (17.6%) and 594 (21%) indicating they often tried or cooked foods seen in mukbang or cookbang (χ^2^ = 20.93, *p* < .001). Among female students, 4,901 (63.4%) who rarely watched reported no impact, while 1,152 (30%) who watched almost daily reported a significant impact on trying or cooking foods seen in these broadcasts (χ^2^ = 27.27, *p* < .001), indicating a more substantial effect on females with higher viewing frequencies.

**Table 4:** Effects on eating habits by number of mukbang and cookbang watching (*n* = 36,026)

Categories	Male				Female			
	Rare	Often	Almost everyday	χ2 (*p*)	Rare	Often	Almost everyday	χ2 (*p*)
None	4,933 (71.8)	4,256 (63.2)	1,693 (59.0)	20.93 (< 0.001)	4,901 (63.4)	4,410 (54.5)	1,923 (49.1)	27.27 (< 0.001)
eat quickly	190 (2.6)	222 (3.0)	105 (3.2)		115 (1.4)	139 (1.7)	71 (1.7)	
eat a lot	280 (4.2)	406 (5.9)	174 (6.2)		185 (2.6)	182 (2.3)	128 (3.2)	
Eating or cooking food from Mukbang or Cookbang	894 (13.1)	1,182 (17.6)	594 (21.0)		1,608 (21.4)	2,200 (27.7)	1,152 (30.0)	
Eat snacks or late-night snacks other than meals	367 (5.5)	465 (7.0)	167 (5.6)		509 (6.6)	653 (8.2)	301 (8.0)	
Eating spicy, salty, sweet, greasy, etc.	182 (2.7)	215 (3.3)	137 (4.9)		349 (4.6)	441 (5.6)	297 (7.9)	

BMI, Body Mass Index

### BMI, body perception, and body image distortion by watching mukbang and cookbang among male and female adolescents

Body mass index, body image perception, and body image distortion relative to the frequency of mukbang and cookbang viewing are outlined in Table 5. For male students, significant differences were found in BMI, body perception, and body image distortion according to mukbang and cookbang viewing frequency. For female students, significant differences were observed except for body image distortion, with daily watching females more likely to have an over-perception compared to under-perception, contrary to males who had more under-perception.

**Table 5:** Body mass index, body image perception and body image distortion by number of mukbang and cookbang watching (*n* = 50,455)

Variables	Categories	Male’s Frequency of watching Mukbang and Cookbang					Female’s Frequency of watching Mukbang and Cookbang				
		None	Rare	Often	Almost everyday	χ2 (*p*)	None	Rare	Often	Almost everyday	χ2 (*p*)
BMI	Low weight	784 (8.6)	491 (7.2)	447 (7.1)	197 (6.9)	7.14 (< 0.001)	564 (11.3)	701 (9.6)	775 (10.0)	383 (10.1)	2.52 (0.008)
	Normal weight	6,223 (67.5)	4,503 (66.2)	4,437 (66.0)	1,785 (63.0)		3,746 (73.8)	5,605 (73.0)	5,898 (73.9)	2,827 (73.7)	
	Overweight	932 (10.0)	759 (10.8)	754 (11.0)	338 (11.1)		395 (7.2)	643 (8.4)	604 (7.4)	325 (7.8)	
	Obesity	1,350 (13.9)	1,093 (15.7)	1,108 (15.9)	550 (18.9)		435 (7.7)	718 (9.0)	748 (8.7)	337 (8.4)	
Body image perception	Under perception	2,602 (28.3)	1,798 (26.5)	1,710 (24.8)	669 (23.3)	7.71 (< 0.001)	785(15.5)	1,100(14.4)	1,110(13.9)	522(13.2)	3.05 (0.006)
	Equal perception	5,530 (59.3)	4,156 (60.2)	4,187(62.6)	1,783 (61.8)		3,289(63.6)	4,971(64.8)	5,220(64.9)	2,451(63.5)	
	Over perception	1,157 (12.4)	892 (13.4)	849 (12.7)	418 (14.8)		1,066(20.9)	1,596(20.8)	1,695(21.2)	899(23.3)	
Body image distortion	No	5,530 (59.3)	4,156 (60.2)	4,187 (62.6)	1,783 (61.8)	6.26 (< 0.001)	3,289(63.6)	4,971 (64.8)	5,220 (64.9)	2,451 (63.5)	1.31 (0.271)
	Yes	3,759 (40.7)	2,690 (39.8)	2,559 (37.4)	1,087 (38.2)		1,851 (36.4)	2,696 (35.2)	2,805 (35.1)	1,421 (36.5)	

### Predictive factors for watching mukbang and cookbang among male and female adolescents

Predictive factors influencing the likelihood of watching mukbang and cookbang among adolescents are analyzed in Table 6. In a three-stage model analysis, no significant differences were observed across all stages for male students. However, living with relatives or boarding increased the likelihood of watching mukbang and cookbang by 1.56 times, and normal sleep compared to sufficient sleep showed a 1.25 times higher likelihood of viewing, both statistically significant. Being obese increased the likelihood by 1.14 times, whereas being underweight showed a reduced likelihood of 0.82 times, both significant. Under perception compared to equal perception was significantly lower at 0.87 times.

**Table 6:** Predictive factors Based on the watching of mukbang and cookbang in adolescent

		Male						Female					
Variables	Categories	Model I		Model II		Model III		Model I		Model II		Model III	
		OR	95% CI	OR	95% CI	OR	95% CI	OR	95% CI	OR	95% CI	OR	95% CI
**Socio-demographic**													
Grade of school (ref = High school)	Middle school	1.12^***^	1.05–1.19	1.15^***^	1.08–1.22	1.15^***^	1.08–1.23	1.10^*^	1.01–1.19	1.11^**^	1.02–1.20	1.11^**^	1.03–1.21
Academic performance (ref = High)	Low	1.68^***^	1.54–1.83	1.66^***^	1.52–1.81	1.64^***^	1.50–1.78	2.00^***^	1.79–2.24	1.97^***^	1.76–2.21	1.96^***^	1.74–2.19
	Middle	1.31^***^	1.21–1.41	1.30^***^	1.21–1.41	1.30^***^	1.20–1.39	1.51^***^	1.37–1.67	1.50^***^	1.36–1.65	1.49^***^	1.36–1.64
Socioeconomic status (ref = High)	Low	1.19^**^	1.06–1.33	1.14^*^	1.02–1.29	1.15^*^	1.03–1.29	1.01	0.86–1.18	1.10	0.83–1.14	0.96	0.82–1.13
	Middle	1.12^**^	1.04–1.22	1.10^*^	1.01–1.20	1.10^*^	1.01–1.20	1.13	1.00-1.28	1.11	0.98–1.25	1.10	0.97–1.25
Living type (ref = With family)	Boarding house/relatives	1.54^***^	1.19–1.99	1.57^***^	1.21–2.03	1.56^***^	1.21–2.02	0.98	0.71–1.36	1.00	0.72–1.38	0.99	0.71–1.38
	Dorms	1.01	0.81–1.26	1.01	0.81–1.25	1.00	0.81–1.25	0.90	0.71–1.14	0.90	0.71–1.15	0.90	0.71–1.14
**Psychological**													
Perceived fatigue recovery by sleep (ref = Enough)	Not enough			1.17^***^	1.09–1.25	1.18^***^	1.10–1.26			1.19^***^	1.09–1.29	1.19^***^	1.09–1.29
	Moderate			1.25^***^	1.17–1.34	1.25^***^	1.17–1.34			1.24^***^	1.13–1.37	1.25^***^	1.13–1.37
Perceived Stress (ref = Low)	High			1.25^***^	1.16–1.35	1.24^***^	1.15–1.34			1.23^***^	1.13–1.35	1.23^***^	1.13–1.35
	Moderate			1.26^***^	1.17–1.35	1.25^***^	1.17–1.34			1.21^***^	1.10–1.33	1.21^***^	1.10–1.33
**Body image related**													
BMI (ref = Normal weight)	Obesity					1.14^***^	1.06–1.22					1.08	0.96–1.22
	Over weight					1.09^*^	1.00-1.19					1.04	0.92–1.18
	Low weight					0.82^***^	0.74–0.92					0.87^**^	0.78–0.97
Body image perception (ref = Equal perception)	Under perception					0.87^***^	0.81–0.92					0.90^*^	0.82–0.98
	Over perception					1.05	0.97–1.14					0.99	0.90–1.07

CI = confidence interval; OR = odds ratio; ref = reference group; ^*^p < .05, ^**^p < .01, ^***^p < .001

For female students, no significant differences were observed across the stages. Students with lower academic performance had a 1.96 times higher risk of watching compared to those with higher performance, a higher risk than male students. Underweight status significantly reduced the likelihood of viewing to 0.87 times, with no significant difference observed in obese and overweight categories. Under perception was significantly lower at 0.90 times compared to equal perception, with over perception not showing significant differences.

## Discussion

This study explored the impact of the recently popular mukbang and cookbang on Korean adolescents, particularly examining factors related to body perception associated with these trends. Given that mukbang and cookbang have been shown to influence eating habits negatively in adults [11], it can be argued that their impact on adolescents, who may have less self-regulation, is even more significant. Therefore, the significance of this study lies in identifying the predictive factors of mukbang and cookbang consumption based on national data representing Korean adolescents, aligning with current trends.

Firstly, the analysis of the frequency of watching mukbang and cookbang revealed a high proportion of adolescents frequently engaging with these types of content. Notably, the combined percentage of female students who watch mukbang and cookbang three to four times a week or daily amounted to 28.4%, which is higher than those who reported never watching these broadcasts. While there is a lack of specific research on mukbang and cookbang for an exact comparison, numerous studies have demonstrated the negative impact of media, games, and social networking services (SNS) on academics, psychology, and problematic behaviors among adolescents [12]. Given that watching mukbang and cookbang is a part of media consumption, it is anticipated to potentially have adverse effects on adolescents, similar to previous research findings. However, due to the current lack of evidence, it is considered necessary to focus research on the psychological, behavioral problems, and health behaviors of adolescents in relation to mukbang and cookbang consumption.

Analyzing the impact of watching mukbang and cookbang on eating habits, it was notably found that 30% of female students who watch these broadcasts almost daily reported eating and cooking similarly to the content viewed. Additionally, the combined percentage of those influenced to consume snacks, late-night meals, or spicy and stimulating foods was also high at 15.9%. These findings indicate a significant relationship between frequent consumption of mukbang and cookbang content and eating disorders in adults [13], suggesting the need for awareness regarding the frequent viewing of such content. Given that adolescents require proper nutrition intake more so than adults to avoid health deterioration due to inappropriate eating habits [14], special attention is needed for managing the eating habits of adolescents who frequently watch mukbang and cookbang.

The analysis of predictive factors based on whether adolescents watch mukbang and cookbang showed that psychological factors such as fatigue and stress were significant risk factors for both male and female adolescents. This means that adolescents who watch mukbang and cookbang tend to have insufficient recovery from fatigue through sleep and also experience higher levels of stress. Similar to recent research findings, the addictive watching of mukbang and cookbang has been significantly correlated with depression, anxiety, and stress among adults, suggesting that watching these broadcasts could be used as a maladaptive coping mechanism [15]. Particularly for adolescents, who are more affected by poor sleep quality and high stress levels than adults, these conditions can impact academic performance and various other factors [16, 17]. Therefore, future research should clarify the causal relationships of the psychological factors among students who watch mukbang and cookbang, necessitating more specific studies in this area.

Additionally, the analysis revealed that obese male students have a higher risk of watching mukbang and cookbang compared to those with normal weight, and this was statistically significant. In contrast, for female students, only those who are underweight showed a lower risk factor for watching mukbang and cookbang compared to normal weight, indicating that, excluding underweight female students, BMI does not significantly affect the likelihood of watching mukbang and cookbang among girls. However, for boys, being obese increases the risk of engaging with these types of content. Frequent viewing of mukbang and cookbang can lead to increased appetite and decreased physical activity, raising the likelihood of obesity [18]. Therefore, it is essential to further investigate the relationship between watching mukbang and cookbang and obesity among adolescents to develop appropriate strategies.

In terms of body perception, it was predicted that male students with under perception are about 0.87 times less likely to watch mukbang and cookbang, which was statistically significant, and similar results were observed for female students. This suggests that students who perceive themselves as thinner than normal or perceive themselves as normal when obese might experience less stress related to their body image or engage in less comparative behavior [19]. Such characteristics could lead to less frequent watching of mukbang and cookbang among these students, with this result being particularly significant among male students. Given that female students tend to have less accurate body perception and higher dissatisfaction with their body image compared to male students [20], future longitudinal studies analyzing the relationship between body perception, body image distortion, and the viewing of mukbang and cookbang are necessary.

Although previous research suggests that living arrangements can influence overall media consumption and risk behaviors such as smartphone addiction [21] our study found that these factors do not significantly impact the specific behavior of watching mukbang and cookbang among adolescents. This indicates that the consumption of mukbang and cookbang might be influenced more by individual preferences or social trends rather than by traditional family structures or living conditions [11]. It suggests that the contents of mukbang and cookbang, which often includes elements of entertainment and social interaction, might appeal to adolescents independently of their living arrangements. Therefore, while traditional factors like family presence may influence general media habits, they do not appear to dictate specific patterns such as the consumption of mukbang and cookbang. This distinction underscores the need for further research to explore what unique factors drive adolescents towards these particular types of media content.

This study, being a secondary data analysis based on cross-sectional data collection, has limitations in explaining causal relationships between variables. Therefore, it is recommended to conduct longitudinal studies in the future to elucidate the changes and causal relationships among variables related to watching mukbang and cookbang during the growth process of adolescents. Nonetheless, this study is significant in that it analyzed the frequency of watching mukbang and cookbang and its impact on eating habits, BMI, and factors related to body perception, distinguishing between male and female adolescents. Based on the findings of this study, further research is needed to analyze the causal relationships of related variables specifically among adolescents who watch mukbang and cookbang. It is suggested that future interventions should be developed to address the eating habits, psychological aspects, and body perception related factors among these adolescents.

## Conclusion

This study utilized the Youth Health Behavior Online Survey to analyze the general characteristics and psychological factors, particularly body perception, of adolescents who watch the currently popular mukbang and cookbang. A significant finding of this research is the high frequency of adolescents watching mukbang and cookbang content, which has been discovered to negatively affect the quality of sleep and stress levels among adolescents, as well as being associated with body perception.

## Data Availability

Please contact the corresponding author for data availability. This study was conducted using data from the 2022 Youth Health Risk Behavior web-base survey (URL: http://www.kdca. go.kr/yhs/).
